# Diagnostic Criteria for Adult-Onset Periodic Fever, Aphthous Stomatitis, Pharyngitis, and Cervical Adenitis (PFAPA) Syndrome

**DOI:** 10.3389/fimmu.2017.01018

**Published:** 2017-08-24

**Authors:** Luca Cantarini, Antonio Vitale, Ludovico Luca Sicignano, Giacomo Emmi, Elena Verrecchia, Isabella Patisso, Lucia Cerrito, Claudia Fabiani, Gabriele Cevenini, Bruno Frediani, Mauro Galeazzi, Donato Rigante, Raffaele Manna

**Affiliations:** ^1^Research Center of Systemic Autoinflammatory Diseases, Behçet’s Disease and Rheumatology-Ophthalmology Collaborative Uveitis Center, Department of Medical Sciences, Surgery and Neurosciences, University of Siena, Siena, Italy; ^2^Periodic Fevers Research Center, Institute of Internal Medicine, Fondazione Policlinico Universitario A. Gemelli, Università Cattolica Sacro Cuore, Rome, Italy; ^3^Department of Experimental and Clinical Medicine, University of Florence, Florence, Italy; ^4^Department of Ophthalmology, Humanitas Research Center, Rozzano, Italy; ^5^Department of Biotechnology, Chemistry and Pharmacy, University of Siena, Siena, Italy; ^6^Institute of Pediatrics, Fondazione Policlinico Universitario A. Gemelli, Università Cattolica Sacro Cuore, Rome, Italy

**Keywords:** PFAPA syndrome, autoinflammatory disease, differential diagnosis, diagnostic criteria, adults, fever of unknown origin

## Abstract

**Objective:**

To identify a set of variables that could discriminate patients with adult-onset periodic fever, aphthous stomatitis, pharyngitis, and cervical adenitis (PFAPA) syndrome from subjects with fever of unknown origin (FUO).

**Methods:**

We enrolled 74 adults diagnosed with PFAPA syndrome according to the currently used pediatric diagnostic criteria and 62 additional patients with FUO. After having collected clinical and laboratory data from both groups, univariate and multivariate analyses were performed to identify the variables associated with PFAPA diagnosis. Odds ratio (OR) values, their statistical significance, and corresponding 95% confidence interval (CI) were evaluated for each diagnostic factor both at the univariate and multivariate analyses. Diagnostic accuracy was evaluated by the area under receiver operating characteristic (ROC) curve, while the leave-one-out cross-validation procedure was used to ensure that the model maintains the same diagnostic power when applied to new data.

**Results:**

According to the multivariate analysis, the clinical variables that discriminated PFAPA patients were: fever episodes associated with cervical lymphadenitis (OR = 92; *p* < 0.0001), fever attacks associated with erythematous pharyngitis (OR = 231; *p* < 0.0001), increased inflammatory markers during fever attacks (OR = 588; *p* = 0.001), and the lack of clinical and laboratory signs of inflammation between flares (OR = 1202; *p* < 0.0001). These variables were considered for a diagnostic model which accounted for their OR values. The diagnostic accuracy of the proposed set of criteria corresponded to an area under ROC curve of 0.978 (95% CI 0.958–0.998), with a model sensitivity and specificity equal to 93.4% (95% CI 87.5–96.5%) and 91.7% (95% CI 82.8–96.7%), respectively.

**Conclusion:**

we have provided herein a set of clinical diagnostic criteria for adult-onset PFAPA syndrome. Our criteria represent an easy-to-use diagnostic tool aimed at identifying PFAPA patients among subjects with FUO with a high-predictive potential, as shown by its very high sensitivity and specificity.

## Introduction

Periodic fever, aphthous stomatitis, pharyngitis, and cervical adenitis (PFAPA) syndrome belongs to the spectrum of multifactorial autoinflammatory diseases (AIDs) and is characterized by spontaneous flares of systemic inflammation characterized by fever and other clinical manifestations, especially cardinal signs described by the PFAPA acronym (1).

To date, the pathogenesis of this syndrome remains still obscure, but studies aimed at assessing immunological mechanisms, also supported by therapeutic evidences (2, 3), have highlighted an abnormal interleukin-1 release in response to many environmental triggers, which associates PFAPA syndrome to other hereditary periodic fever disorders (4, 5). However, unlike other AIDs characterized by recurrent fever attacks, no genetic mutations have been clearly associated with PFAPA syndrome (6, 7).

In addition to fever (often achieving and overcoming 40°C), aphthous stomatitis, pharyngitis, and cervical adenitis, many other clinical manifestations may enrich the clinical framework of PFAPA patients, including abdominal pain, headache, nausea, skin manifestations, and arthralgia (1, 8–10). Inflammatory flares arise every 3–8 weeks with no premonitory symptoms and generally last 3–6 days. Patients are typically healthy between febrile episodes and the overall growth of children affected by this syndrome is not stunted (11, 12). Although during the last decades diagnosis of PFAPA syndrome has been relegated to children aged under 5 years, increasing evidence has recently shown that the disease can also arise in older children as well as during adulthood (2, 3, 9, 10, 13–18). The treatment of PFAPA patients is based on intermittent corticosteroid administration, as patients are generally responsive to a single dose of a corticosteroid given at the onset of febrile flares (1, 12, 14, 19, 20).

No laboratory or instrumental tools are available to support the diagnosis of PFAPA syndrome, which is currently based on the fulfillment of clinical diagnostic criteria. In particular, to date, clinical criteria proposed by Marshall et al. in 1986 (21) and later modified by Thomas et al. in 1999 (11) represent the most used set of criteria in the clinical practice. However, these criteria are tailored on pediatric patients and their application on adults is categorically excluded by the first item that requires the presence of recurrent fever in patients under 5 years of age. In addition, the fifth item imposes the lack of normal growth and development for patients affected, which is not applicable to adult-onset PFAPA patients. In this context, Padeh et al. employed a further set of inclusion criteria valid for both children and adult patients (8). This set included the presence of monthly fever attacks, exudative tonsillitis, possibly oral ulcers, cervical lymph node enlargement, negative throat cultures, and failure of antibiotic treatment during the acute episodes or as prophylactic treatment, while normal growth/development and a rapid response to a single corticosteroid administration were later added as further items (8, 22). However, to the best of our knowledge, no statistical procedures were employed to identify variables useful in discriminating PFAPA patients among subjects presenting with recurrent fever attacks. In addition, recent evidences have proved that erythematous pharyngitis is more typical than sterile exudative pharyngitis in adult-onset PFAPA patients (10). Therefore, the need for a new set of diagnostic criteria for patients experiencing PFAPA syndrome during adulthood has prompted our group to evaluate a set of variables on both clinical and statistical basis that could discriminate such patients from subjects with fever of unknown origin (FUO).

## Materials and Methods

### Patients

Seventy-four consecutive adult patients who had been referred to our Units from September 2007 to December 2016 because of recurrent fever attacks and other clinical manifestations consistent with PFAPA syndrome were classified as suffering from adult-onset PFAPA syndrome (PFAPA group) according to the Marshall criteria modified by Thomas et al. (11, 21), which are the most frequently used diagnostic tool in the clinical practice. As this set of criteria is tapered on pediatric patients, the item requiring a disease onset before the age of 5 was neglected, while the item requiring a normal growth and development was retrospectively applied, as previously made in other studies (9, 10, 13). Two patients out of 74 were included in the PFAPA group despite the lack of symptom-free intervals. In both cases, the patients showed the resolution of fever and of cardinal symptoms as well as the normalization of acute phase reactants. Conversely, the sole arthralgia and myalgia persisted in both cases and were attributed to the presence of concomitant degenerative, not inflammatory musculoskeletal diseases.

Sixty-two additional adult subjects admitted in our Units between September 2016 and March 2017 for recurrent FUO were consecutively enrolled in the study as control group (control group). FUO diagnosis was based on the currently available diagnostic criteria (23). As for patients with adult-onset PFAPA syndrome, any specific disease related to fever or inflammatory manifestations had been ruled out at the time of enrollment in this study. The control group was included into a follow-up protocol aimed at early identify any sign or symptom potentially useful for a prompt specific diagnosis; patients were treated with non-steroidal anti-inflammatory drugs or low-to-high dosage corticosteroids.

### Assessment Parameters

Each patient’s medical record was reviewed for demographic and clinical data. In particular, we looked for the age at disease onset, characteristics of the febrile pattern (peak temperature, duration of flares, frequency of fever episodes per year), clinical manifestations accompanying fever (oral and/or genital aphthosis, exudative and/or erythematous pharyngitis, cervical and/or widespread lymphadenitis, abdominal pain, vomiting, diarrhea, thoracic pain, arthralgia, arthritis, myalgia, urticarial-like rash, maculopapular rash, erysipelas-like rash, erythematous rash, periorbital edema, conjunctivitis, asthenia, and headache), any increase of inflammatory markers (erythrocyte sedimentation rate and/or C-reactive protein and/or serum amyloid A) during attacks and the presence or absence of clinical manifestations and positive laboratory inflammatory markers during fever-free intervals.

None of the patients with adult-onset PFAPA syndrome showed upper respiratory infections, while the throat swab was negative in all cases presenting with pharyngitis or cervical lymphadenitis. Both PFAPA group and control group patients underwent detailed laboratory and instrumental screening tests to rule out potential underlying diseases, such as infections, autoimmune diseases, and malignancies. In all patients enrolled, previous antibiotic therapies administered during flares did not change the progression of clinical manifestations. Monogenic periodic fever syndromes were ruled out by performing molecular analysis of *MEFV, MVK, TNFRSF1A*, and *NLRP3* genes, respectively responsible for familial Mediterranean fever (FMF), mevalonate kinase deficiency, tumor necrosis factor receptor-associated periodic syndrome, and cryopyrin-associated periodic syndrome (CAPS). Moreover, neither PFAPA patients nor subjects included in the control group fulfilled clinical diagnostic criteria for FMF or CAPS as well as for Still’s disease, Schnitzler’s syndrome, and Behçet’s disease (24–33).

The study was approved by the local Ethics Committee of Azienda Ospedaliera Universitaria Senese, Siena (Italy) and each patient provided a written consent for both genetic testing and clinical data processing, in accordance with the Declaration of Helsinki.

### Statistical Analysis

Descriptive statistics are expressed as mean and SD for quantitative variables as well as frequency counts and percentages for quantitative binary variables.

Multivariate stepwise logistic regression analysis was performed to identify, among all possible diagnostic factors (predictive variables), a statistically significant minimum subset of factors with the highest possible accuracy to establish a diagnosis of PFAFA syndrome. In the stepwise process, one independent variable was added to or removed from the discriminant model at each step, on the basis of maximum likelihood-ratio statistics. The process stops when no statistical significant variables can be more entered or removed. We used the leave-one-out (LOO) cross-validation procedure to ensure that the model maintains the same diagnostic power when applied to new data. LOO uses all available data to train and test model: it executes a number of training sessions equal to the sample size (*N*) and in each of them it classifies each patient (LOO testing case) in turn by using all other patients as training set.

Diagnostic accuracy was evaluated by the area under receiver operating characteristic (ROC) curve (AUC) along with its 95% confidence interval (95% CI). Model sensitivity and specificity together with their 95% CIs were also estimated by selecting a probability threshold giving comparable sensitivity and specificity values, along with their 95% CIs. The Hosmer–Lemeshow goodness-of-fit test was used to evaluate model calibration, that is its prognostic ability.

Finally, the odds ratio (OR), its statistical significance, and corresponding 95% CI were evaluated for each diagnostic factor, taken singularly (univariate analysis), and for the model selected factors, taken together (multivariate analysis). The SPSS software, version 10, was used for all statistical computations, always considering a significance level of 95% (*p* value < 0.05).

## Results

Both patients with adult-onset PFAPA syndrome and subjects belonging to the control group experienced a disease onset over the age of 16. Specifically, the mean age at disease onset was 26.55 ± 10.03 years for PFAPA patients and 27.94 ± 17.67 for those with FUO. Table 1 summarizes demographic and clinical features of patients enrolled.

**Table 1:** Demographic and clinical features of patients diagnosed with periodic fever, aphthous stomatitis, pharyngitis, and cervical adenitis (PFAPA) syndrome (PFAPA group) and patients with fever of unknown origin (control group).

	PFAPA group	Control group
Age (years)	34.00 ± 11.86	40.56 ± 16.45
Males (%)/females (%)	48 (64.9)/26 (35.1)	23 (37.1)/39 (62.9)
Age at disease onset (years)	26.55 ± 10.03	27.94 ± 17.67
Mean temperature at attacks (°C)	39.31 ± 0.92	38.9 ± 1.02
Attacks per year	15.2 ± 8.44	9.45 ± 7.03
**Duration of flares**		
≤2 days	2 (2.7%)	14 (22.6%)
3–5 days	46 (62.2%)	12 (18.2%)
6–9 days	9 (12.2%)	8 (12.9%)
≥10 days	11 (14.9%)	22 (35.5%)
**PFAPA cardinal symptoms during attacks**		
Pharyngitis	70 (94.6%)	39 (62.9%)
Cervical lymphadenitis	61 (82.4%)	14 (22.6%)
Oral aphthosis	48 (64.9%)	21 (33.9%)
**Other associated symptoms during attacks**		
Generalized lymphadenitis	4 (5.4%)	14 (22.6%)
Asthenia	62 (83.8%)	51 (82.3%)
Abdominal pain	33 (44.6%)	20 (32.3%)
Diarrhea and/or vomiting	16 (21.6%)	13 (21%)
Thoracic pain	13 (17.6%)	24 (38.7%)
Arthralgia	53 (71.6%)	42 (67.7%)
Arthritis	11 (14.9%)	16 (25.8%)
Myalgia	47 (63.5%)	37 (59.7%)
Urticaria-like rash	4 (5.4%)	10 (16.1%)
Erythematous rash	9 (12.2%)	0 (0.0%)
Erysipelas-like rash	0 (0.0%)	3 (4.8%)
Maculo-papular rash	3 (4.1%)	9 (14.5%)
Periorbital edema	6 (8.1%)	5 (8.1%)
Conjunctivitis	8 (10.8%)	18 (29%)
Headache	43 (58.1%)	37 (59.7%)
Genital aphthosis	3 (4.1%)	2 (3.2%)
**Increased inflammatory markers during attacks**	72 (97.3%)	46 (74.2%)
**Symptom-free intervals**	72 (97.3%)	33 (53.2%)

*Quantitative data are referred as mean ± SD values; qualitative data are reported as frequency counts and percentages*.

Univariate analysis performed on patients with PFAPA syndrome and subjects with FUO recognized clinical variables positively or negatively associated with PFAPA syndrome by an OR significantly different from 1, i.e., with a 95% CI not including 1.0. The results of univariate analysis are summarized in Table 2.

**Table 2:** Results of univariate logistic regression analysis performed on adult-onset periodic fever, aphthous stomatitis, pharyngitis, and cervical adenitis (PFAPA) patients and subjects with fever of unknown origin by evaluating clinical manifestations described in both groups.

Clinical variable	*p*-Value	Sensitivity (%)	Specificity (%)	OR	95% CI
Age	0.010	47.5	81.1	0.968	0.944–0.992
Age at onset	0.591	1.7	98.6	0.994	0.971–1.017
Frequency of flares	0.001	51.1	81.2	1.108	1.046–1.174
**Duration of flares**					
≤2 days	0.003	23.0	97.1	0.099	0.021–0.455
3–5 days	<0.0001	80.0	65.7	7.667	3.437–17.102
6–9 days	0.936	0.0	100	0.959	0.345–2.664
≥10 days	0.006	37.3	84.3	0.314	0.136–0.721
Increased inflammatory markers during attacks	0.065	11.5	97.3	4.696	0.909–24.268
Symptom-free intervals	<0.0001	45.9	97.3	30.545	6.867–135.878
Oral aphthosis	0.001	65.6	64.9	3.516	1.726–7.166
Pharyngitis	0.010	64.5	86.5	1.943	1.169–3.229
Erythematous pharyngitis	<0.0001	91.9	78.4	41.325	14.194–120.315
Exudative pharyngitis	0.005	27.4	91.9	0.234	0.086–0.637
Laterocervical lymphadenitis	<0.0001	76.3	82.4	15.082	6.463–35.199
Generalized lymphadenitis	0.006	22.6	94.6	0.196	0.061–0.631
Asthenia	0.813	0.0	100	1.114	0.454–2.736
Abdominal pain	0.163	67.2	44.6	1.650	0.816–3.337
Diarrhea/vomiting	0.965	0.0	100	1.019	0.446–2.326
Thoracic pain	0.006	39.3	82.4	0.329	0.149–0.723
Arthralgia	0.624	0.0	100	1.202	0.577–2.504
Arthritis	0.104	26.2	85.1	0.491	0.208–1.158
Myalgia	0.647	0.0	100	1.176	0.588–2.354
Skin rash	0.531	0.0	100	0.776	0.350–1.718
Urticaria-like rash	0.047	16.4	94.6	0.291	0.087–0.982
Erythematous rash	0.999	0.0	100	NE	0.000–NE
Maculo-papular rash	0.041	14.8	95.9	0.244	0.063–0.946
Erysipelas-like rash	0.999	4.9	100	0.000	0.000–NE
Periorbital edema	0.993	0.0	100	1.006	0.292–3.469
Conjunctivitis	0.009	29.0	89.2	0.296	0.119–0.741
Headache	0.764	0.0	100	0.900	0.451–1.795
Genital aphthosis	0.787	0.0	100	1.286	0.208–7.952

*NE, not evaluable; CI, confidence interval; OR, odds ratio*.

As reported in Table 3, according to multivariate analysis performed on the two groups of patients, clinical variables that showed a statistical significant (*p* < 0.05) discriminant power to identify PFAPA patients were: recurrent fever accompanied by cervical lymphadenitis (OR = 92), recurrent fever with concomitant erythematous pharyngitis (OR = 231), increased inflammatory markers during attacks (OR = 588), and symptom-free intervals corresponding to the lack of clinical manifestations and laboratory abnormalities between flares (OR = 1,202). These variables were then considered for a diagnostic model that accounts for their OR values. In particular, the occurrence of symptom-free intervals and the increase of inflammatory markers during attacks, which have higher OR values, represent mandatory items in the proposed diagnostic model. Conversely, on the basis of their lower OR values, only one between fever associated with erythematous pharyngitis and fever with cervical lymphadenitis is required for the diagnosis of PFAPA syndrome. Table 4 shows the resulting set of criteria proposed in this study.

**Table 3:** Estimations derived from multivariate logistic regression analysis performed on adult periodic fever, aphthous stomatitis, pharyngitis, and cervical adenitis (PFAPA) patients and patients with fever of unknown origin, representing the control group.

Clinical variable	*p*-Value	OR	95% CI
Erythematous pharyngitis	<0.0001	231	14.463–3,715.288
Cervical lymphadenitis	<0.0001	92	8.865–953.279
Increased inflammatory markers during attacks	0.001	588	3,534.3–40,879.463
Symptom-free intervals	<0.0001	1202	12.631–27,937.885

*CI, confidence interval; OR, odds ratio*.

**Table 4:** Clinical diagnostic criteria resulting from the multivariate analysis.

Diagnostic criteria for adult-onset periodic fever, aphthous stomatitis, pharyngitis, and cervical adenitis (PFAPA) syndrome
Recurrent fever accompanied by (a) Erythematous pharyngitis and/or (b) Cervical lymphadenitis
Increased inflammatory markers during attacks
Symptom-free intervals

*Diagnostic items accounted for their odds ratio (OR) values: variables with a higher OR value (increased inflammatory markers during attacks and symptom-free intervals) were established as *mandatory* in the final diagnostic model; conversely, on the basis of a lower OR value, only one item between erythematous pharyngitis during fever and cervical lymphadenitis during fever is required for the diagnosis of PFAPA syndrome. These diagnostic criteria should be applied on patients aged at least 16 years and after having excluded infective, autoimmune, and neoplastic diseases as well as monogenic autoinflammatory diseases (AIDs) and febrile polygenic AIDs. In addition, throat swab performed during fever have to be negative and antibiotic therapy ineffective*.

**Figure 1:**
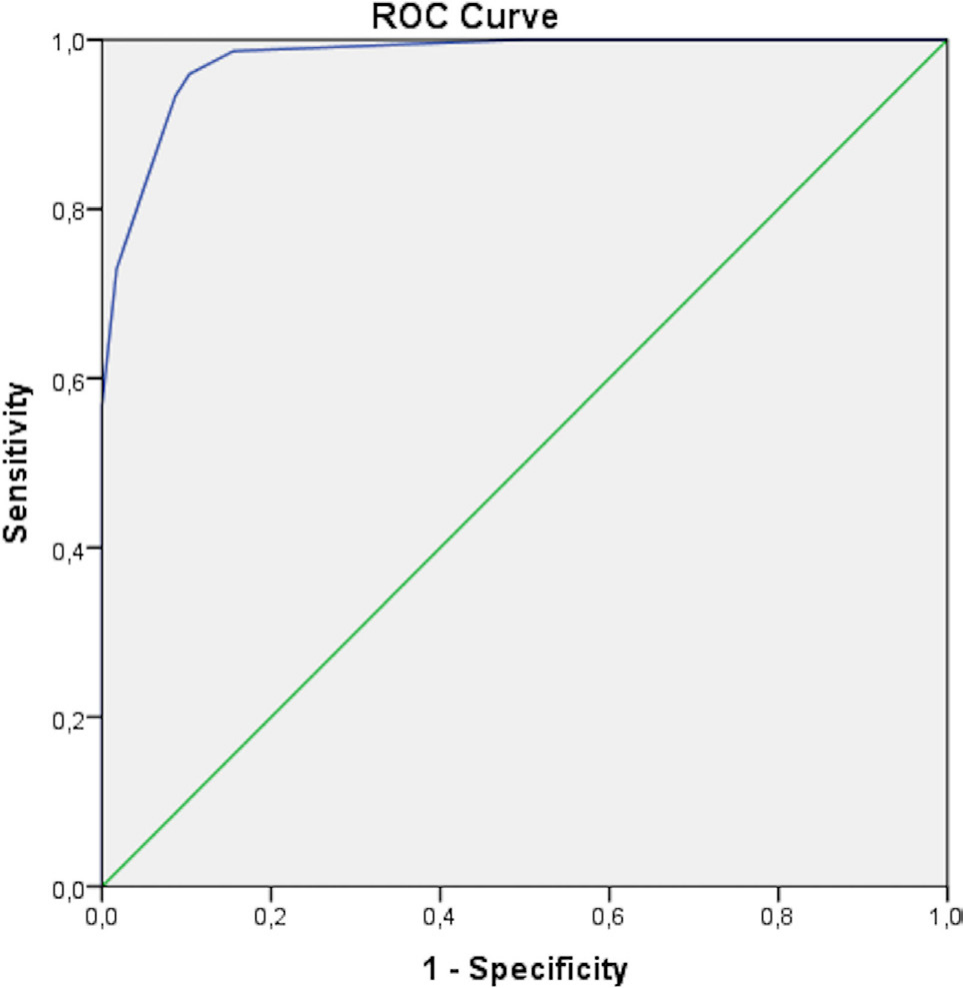
Receiver operating characteristic (ROC) curve obtained for adult periodic fever, aphthous stomatitis, pharyngitis, and cervical adenitis (PFAPA) patients and subjects with fever of unknown origin as control group. The area under curve is of 0.978 (95% CI 0.958–0.998), corresponding to sensitivity of 93.4% (95% CI 87.5–96.5%) and specificity of 91.7% (95% CI 82.8–96.7%) for the proposed diagnostic model.

The diagnostic accuracy of the proposed diagnostic criteria corresponded to an AUC of 0.978 (95% CI 0.958–0.998), with sensitivity and specificity equal to 93.4% (95% CI 87.5–96.5%) and 91.7% (95% CI 82.8–96.7%), respectively. Figure 1 represents the ROC curve assessing the performance of the criteria for our PFAPA patients and the control group with FUO.

## Discussion

Despite the increasing evidence on the possible delayed onset of PFAPA syndrome during adulthood, current diagnostic criteria are tailored on pediatric patients (34) and their application on adults requires specific adjustments not yet validated. On this basis, we looked for clinical variables that can identify patients with adult-onset PFAPA syndrome among patients presenting with FUO. Therefore, we analyzed the occurrence of inflammatory features in patients with a clinical picture consistent with adult-onset PFAPA syndrome, being excluded all the known causes of recurrent fever, as well as in patients consecutively visited in our Units because of FUO during a 6-month period. Multivariate analysis allowed to identify a set of clinical variables capable of discriminating adult-onset PFAPA patients. These variables were then rearranged into a diagnostic model in which items with a higher OR were considered mandatory for the diagnosis of PFAPA, while just one out of the two variables with a lower OR value had to be fulfilled.

Noteworthy, these proposed diagnostic criteria should be applied after having ruled out the known causes of fever in terms of infective, autoimmune, and neoplastic diseases. Monogenic AIDs should be also excluded on the basis of clinical presentation, as required by the clinical classification criteria recently proposed by Federici et al. to drive genetic analysis for patients with periodic fevers (35). According to Federici et al., we have also reported that the diagnosis of monogenic AIDs in adulthood is not unworkable when patients’ symptoms are carefully classified (36, 37). Therefore, a correct evaluation of the patients’ clinical picture integrated by familiar and laboratory data may allow the identification of adult-onset monogenic AIDs by specifically performing genetic testing. In addition to this, specific clinical diagnostic and classification criteria, when available, should also be applied to preventively recognize both monogenic (i.e., FMF and CAPS) and multifactorial AIDs (i.e., Behçet’s disease, Still’s disease, and Schnitzler’s disease) (24–33). Moreover, our present criteria should not be applied in patients with positive throat swab during fever episodes and in patients responsive to antibiotics, as for previous diagnostic and classification criteria (11, 22).

Among the cardinal signs of PFAPA syndrome, the occurrence of recurrent fever with erythematous pharyngitis represented the variable most strongly associated with diagnosis of PFAPA syndrome in adulthood, while exudative pharyngitis and oral aphthosis during attacks were not included in the model. Accordingly, we had previously found that the exudative form of pharyngitis is almost rare in patients with a delayed onset of PFAPA syndrome (9, 10), while univariate analysis performed in this study even highlights a protective role of exudative pharyngitis against the diagnosis of PFAPA syndrome, further remarking a less important role of this clinical manifestation in adults. In relation to lymph node involvement, the specific observation of cervical lymphadenitis was strongly correlated to PFAPA syndrome both when considered individually and at the overall multivariate assessment. Conversely, at the univariate analysis, generalized lymphadenitis represented a variable tending to exclude the diagnosis of PFAPA syndrome in adults.

Regarding oral aphthosis, although significantly discriminant when considered singularly, it was not included into the multivariate model as its diagnostic information resulted to be absorbed by “recurrent fever accompanied by erythematous pharyngitis” and “symptom-free intervals.” Therefore, most patients with oral aphthosis also presented at least one out of these two variables included in the model, thus minimizing the diagnostic value of oral aphthosis as an additional item. Interestingly, according with our results, Padeh had already suggested oral ulcers just as “possible” among the classification items proposed in 2005 (22). Furthermore, other authors have also highlighted that oral aphthosis is less frequently encountered in adult-onset PFAPA patients than among children (9, 11, 14). These observations seem to corroborate that aphthous stomatitis, although important for clinical evaluation, does not necessarily have to be included for diagnostic purposes in adults.

In relation to non-cardinal symptoms, beyond generalized lymphadenitis, also thoracic pain, conjunctivitis, maculopapular, and urticarial-like skin rash appeared to be protective factors against PFAPA diagnosis when considered individually. Consequently, the observation of these manifestations in patients with a suspected PFAPA syndrome should call for caution before assigning the diagnosis. In addition, univariate analysis shows that PFAPA syndrome is mostly connected with a fever duration ranging between 2 and 5 days, while fever attacks lasting less than 48 h and longer than 10 days should point to other diagnoses than PFAPA syndrome.

Although results obtained by univariate analysis are clinically interesting and potentially useful to identify or exclude adult-onset PFAPA syndrome, we aimed at creating a set of diagnostic criteria easy to be applied in the clinical practice and reproducible for further studies. Therefore, we deliberately avoided a longer list of diagnostic items as well as concomitant exclusion criteria, without decreasing the predictive potential of the model. Indeed, as demonstrated by the very high level of sensitivity and specificity obtained at ROC analysis, 93.4% of all patients fulfilling the diagnostic criteria would be correctly identified as having PFAPA syndrome and only 8.3% (100% − specificity) would be incorrectly classified as PFAPA patients.

In our casuistry, a male preponderance was observed among patients with adult-onset PFAPA syndrome, while the number of females was higher in the control group. Looking at the past data on adult-onset PFAPA syndrome, a gender imbalance was not clearly observed as the male/female ratio was 1 according to a review evaluating all the cases published until 2015 (38). However, more recently, we have already reported 30 patients characterized by a male preponderance (9). As this trend has been confirmed again in this study, the male preponderance could represent a non-random finding. Nevertheless, future observational studies are required to clarify whether the higher number of males is a stochastic event related to the consecutive enrollment of patients or a specific feature of the disease.

Of note, we did not take into account the complete response to a single dose of corticosteroid as a possible diagnostic item to provide a set of criteria immediately applicable at the first clinical assessment, also in patients never treated with steroids. In addition, the complete resolution of flares after a single-steroid administration has proved to be less pronounced in adults than among pediatric patients. In this regard, we have recently highlighted that 98.8% of 85 pediatric PFAPA patients and only 88.2% out of 17 adult patients with PFAPA syndrome experienced total resolution of flares after a single-corticosteroid administration (10). Since this is probably explained by inadequate corticosteroid dosages in adults, *ad hoc* dosage trials should be conducted on late-onset PFAPA patients before including this variable as an additional diagnostic item.

Although we performed genetic testing in all patients to exclude subjects carrying mutations in genes related to the most frequent monogenic AIDs, we did not perform a testing for myeloid restricted somatic mutations that have recently been described in adult patients and could explain autoinflammatory manifestations in some cases (39–41). This represents a potential limit of the genetic screening strategy adopted in our cohort of patients. Also, the sample size of our study is relatively small due to the rarity of adult-onset PFAPA syndrome. Nevertheless, we have reported herein the largest cohort of patients ever described, adequate for performing a reliable statistic computation aimed at creating diagnostic criteria.

Our diagnostic criteria have been tested on adult patients and should be applied only to subjects aged at least 16 years. Their ability in differentiating adult-onset PFAPA patients from patients with late-onset monogenic AIDs could be tested in future studies.

In conclusion, we provide a set of clinical diagnostic criteria focused on adult patients presenting with suspected adult-onset PFAPA syndrome. They have been designed as an easy-to-use diagnostic tool aimed at identifying PFAPA patients from subjects with FUO with a high-predictive potential as shown by its very high sensitivity and specificity.

## Ethics Statement

The study was approved by the local Ethics Committee of Azienda Ospedaliera Universitaria Senese (AOUS), Siena (Italy) and each patient provided a written consent for both genetic testing and clinical data processing, in accordance with the Declaration of Helsinki.

## Author Contributions

LCa and AV designed the study; LCa and RM finally revised the manuscript; LCa, RM, AV, GE, DR, LLS, GE, EV, IP, LCe, CF, BF, and MG final approval of the manuscript; LCa, AV, and DR drafting of the manuscript; GC and AV data analysis; LLS, GE, EV, IP, LCe, CF, BF, and MG patients enrollment, follow-up of the patients, and data collection.

## Conflict of Interest Statement

We declare that the work was conducted in the absence of any commercial or financial relationship that could be construed as a potential conflict of interest.
